# Influence of process parameters on single-cell oil production by *Cutaneotrichosporon oleaginosus* using response surface methodology

**DOI:** 10.1186/s13068-025-02717-3

**Published:** 2025-11-19

**Authors:** Max Schneider, Felix Melcher, Robert Fimmen, Johannes Mertens, Daniel Garbe, Michael Paper, Marion Ringel, Thomas Brück

**Affiliations:** Werner Siemens-Chair of Synthetic Biotechnology, Technical University of Munich (TUM), TUM School of Natural Sciences, Lichtenbergstr. 4, 85748 Garching, Germany

**Keywords:** *Cutaneotrichosporon oleaginosus*, Oleaginous yeast, Single-cell oil, Response surface methodology, Box–Behnken design, Process optimization, FAMEs, Fatty acid profile

## Abstract

**Background:**

The growing demand for sustainable lipid sources has fostered interest in single-cell oils from oleaginous yeasts as renewable alternatives to plant-derived and fossil-based oils, with applications in food, fuel, and material production. The oleaginous yeast *Cutaneotrichosporon oleaginosus* is of industrial relevance due to its ability to accumulate in excess of 60% (w/w) of its dry cell weight as lipids, while metabolizing a broad range of substrates. However, economic feasibility depends on improving productivity and adapting fatty acid profiles to application requirements.

**Results:**

This study investigated the influence of temperature, pH, and dissolved oxygen concentration (DO) on lipid production and fatty acid composition in *C. oleaginosus* ATCC 20509. A three-level, three-factor Box–Behnken design was applied to assess their effects on lipid titer, oleate lipid titer, and the proportions of saturated and unsaturated fatty acids. Response surface methodology was used to develop quadratic models, identify optimized conditions, and predict fatty acid compositions. Temperature and pH significantly affected both overall lipid titer and degree of saturation. In fed-batch cultivation with consumption-based acetic acid feeding and glucose as the initial carbon source, lipid productivity increased to 0.38 g/L/h under the optimized oleate lipid titer condition (27.6 °C, pH 5.6, 10% DO) and to 0.39 g/L/h under the optimized saturated fatty acid condition (30 °C, pH 7.0, 10% DO), corresponding to 46% and 50% increases compared to literature values (0.26 g/L/h; 28 °C, pH 6.5, 50% DO). The fatty acid profile could thus be precisely modulated by adjusting the process parameters, achieving a difference in the saturation degree of more than 10%. Temperature was identified as the main factor influencing saturation, while pH enabled adjustment of the C16/C18 ratio, resulting in a modulation of palmitic acid fraction within the total triglycerides of up to 13%.

**Conclusion:**

These findings highlight the potential of optimizing cultivation parameters based on reaction surface methodology to simultaneously improve lipid productivity and functionality by tailoring the fatty acid profile to the desired application requirements, without resorting to genetic engineering. Moreover, these insights support a circular bio-based economy by enabling an efficient production of tailored microbial oils as renewable alternatives to plant-derived lipids.

**Graphical Abstract:**

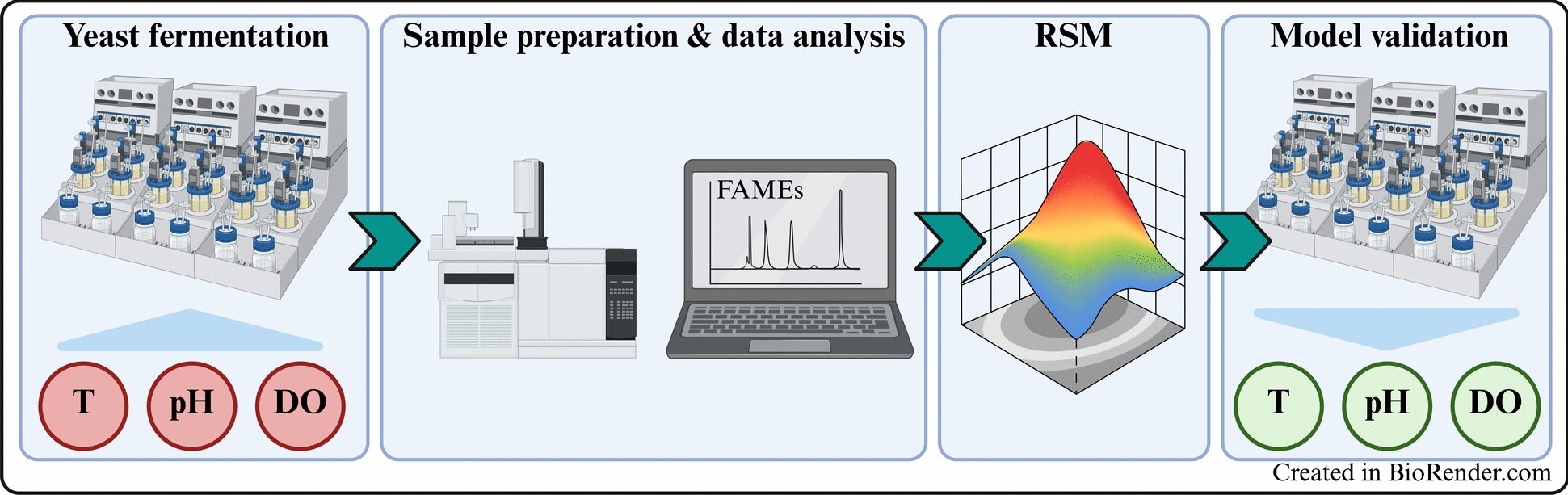

**Supplementary Information:**

The online version contains supplementary material available at 10.1186/s13068-025-02717-3.

## Background

The increasing global demand for sustainable alternatives to conventional plant-derived and fossil-based oils has spurred significant interest in microbial lipids, particularly single-cell oils (SCOs) produced by oleaginous yeasts [1]. These lipids represent a renewable alternative for the production of food ingredients, cosmetics, biofuels, and advanced biomaterials [2]. Among oleaginous yeasts, *Cutaneotrichosporon oleaginosus* has emerged as one of the most potent and industrially relevant oleaginous species, due to its exceptional lipid accumulation capacity, exceeding 60% (w/w) of its dry cell weight in the wild type, as well as its metabolic flexibility and robustness in a wide range of substrates and process conditions [3]. The biotechnological exploitation of oleaginous yeasts for SCO production aligns closely with global sustainability objectives. This includes reducing dependency on land- and water-intensive crops like oil palm, soybean, and rapeseed, while enabling decoupling from seasonal and geopolitical constraints through microbial, scalable processes [4]. Although microbial oils have long been investigated as biofuels, their potential in high-value sectors such as oleochemicals (e.g., lubricants and surfactants) and food technology has gained increasing attention [5–7]. Tailored fatty acid compositions and purity are essential for these applications. Despite its advantages, the economic feasibility of microbial lipid production remains constrained by suboptimal fermentation efficiencies and process variability. To address these limitations, systematic bioprocess development and optimization are essential. Design of experiments (DoE) approaches provide a statistically robust framework for identifying and quantifying the influence of key process parameters, enabling the development of predictive models for performance metrics such as lipid titer, productivity, and fatty acid composition [8]. In contrast to one-factor-at-a-time methodologies, DoE enables the exploration of parameter interactions and nonlinear effects, thereby accelerating process refinement and reducing experimental effort.

While improving fermentation efficiency is crucial for economic viability, the ability to tailor the qualitative properties of microbial oils, especially their fatty acid profiles, is equally important. This fine-tuning through process parameter adjustments provides a versatile and regulation-friendly strategy to meet specific industrial needs without resorting to genetic engineering. Previous studies have shown that the choice of carbon and nitrogen sources, as well as the C/N ratio applied, can influence both lipid productivity and fatty acid profile in oleaginous yeasts [8–11]. Similarly, media-independent process parameters, such as temperature (T), pH, and dissolved oxygen concentration (DO), affect not only microbial growth, but also the productivity and quality of the targeted bioproduct [12–14]. In oleaginous yeasts, these parameters influence biomass and lipid production while also modulating the fatty acid composition of the obtained lipids [15–17]. Variations in the degree of saturation and the fatty acid chain lengths can alter the physicochemical properties of the yeast oil, enabling tailored consumer applications without the need for metabolic engineering or feedstock modifications. This enables the production of SCOs with fatty acid compositions customized to downstream applications, such as oils rich in oleic acid and other monounsaturated fatty acids with high oxidative stability for the food, lubricant, and cosmetic industry, or more saturated oils suitable for biofuel production and as cocoa butter equivalents [6, 7, 18, 19].

In this study, we present a systematic process optimization for SCO production by *C. oleaginosus*, employing a response surface methodology (RSM) to evaluate the impact of cultivation parameters on lipid accumulation. Through a systematic examination of the design space, favorable conditions for enhanced microbial lipid productivity were identified. Furthermore, the obtained data provided valuable insights into how the fatty acid composition can be modulated through process parameters, specifically the degree of saturation and the C16/C18 ratio, to meet targeted application requirements. This work provides a significant contribution to the scalable and sustainable production of SCOs, offering practical insights into yeast oil manufacturing, a key step toward a circular bio-based economy.

## Methods

### Cultivation

#### Strain and preculture

All cultivation experiments were carried out using the oleaginous yeast *Cutaneotrichosporon oleaginosus* ATCC 20509 (DSM-11815), obtained from the Deutsche Sammlung von Mikroorganismen und Zellkulturen (Braunschweig, Germany). For precultures, 100 mL of YPD medium (10 g/L yeast extract, 20 g/L peptone, 20 g/L glucose) in a 500-mL baffled Erlenmeyer flask containing antibiotics (0.1 g/L ampicillin, 0.05 g/L kanamycin) was inoculated with a single colony of *C. oleaginosus* from a YPD agar plate. Cultures were incubated in a rotary shaker at 120 rpm and 28 °C for 3 days.

#### Medium composition

An adapted medium according to Rerop et al. was used for the consumption-based acetic acid fermentation, containing the following components: 30 g/L glucose, 4.5 g/L CH_3_COO∙Na, 3 g/L peptone, 2 g/L yeast extract, 1 g/L urea, 0.9 g/L Na_2_HPO_4_, 2.4 g/L KH_2_PO_4_, 2 g/L MgSO_4_·7H_2_O, 0.5 g/L CaCl_2_·2H_2_O, 0.027 mg/L C_6_H_8_O_7_·Fe·H_3_N, 0.025 mg/L CuSO_4_·5H_2_O, 0.024 mg/L MnCl_2_·6H_2_O, 0.00055 mg/L ZnSO_4_·7H_2_O, and 0.1 mg/L thiamine [20].

#### Fermentation in parallel bioreactors

Fermentations were conducted in a DASbox^®^ 12-fold parallel bioreactor system (Eppendorf AG, Hamburg, Germany) with a maximum working volume of 250 mL. Temperature, pH, and DO were set according to the DoE approach or the validation conditions (see Supporting Information Table S1, S3) and were maintained constant throughout the fermentation run. The starting pH was adjusted using 2 M HCl and 2 M NaOH to ensure comparable initial acetic acid concentrations across all conditions. During fermentation, 50% (v/v) acetic acid was used as consumption-based feed. Antifoam 204 (Merck, Darmstadt, Germany) was used for foam prevention. Initial stirring velocity and air flow were set to 200 rpm and 4.75 sL/h, with an oxygen content of 21%. During fermentation, the dissolved oxygen level was maintained by gradually increasing the stirring speed (max. 650 rpm), oxygen content (max. 100%), and airflow (max. 23 sL/h). For inoculation, 150 mL of fermentation medium was inoculated with 1 mL of the washed preculture to an initial optical density at 600 nm (OD_600_) of 0.5. Samples for determining OD_600_, dry cell weight, sugar concentration, fatty acid composition, and lipid titer were collected over the course of the fermentation.

### Fermentation monitoring and sample preparation

#### ***OD***_***600***_*** measurement***

Cell growth was monitored by measuring OD_600_ using a UV-3100PC spectrophotometer (VWR, Radnor, PA, USA). For each bioreactor, technical triplicates of each sample were diluted with phosphate-buffered saline to obtain OD_600_ values within the linear range (0.1–1.0).

#### Dry cell weight

For dry cell weight (DCW) determination, 3 mL of fermentation broth was transferred to pre-weighed tubes, mixed with an equal volume of ethanol, and centrifuged at 4400 rcf and 20 °C for 12 min. Cell pellets were resuspended in 6 mL 50% (v/v) aqueous ethanol and centrifuged for 1 h at 4400 rcf and 20 °C. The supernatant was discarded, and the samples were frozen and subsequently lyophilized. Each sample was analyzed in technical duplicates.

#### Microscopic pictures

The fermentation broth of each reactor was examined every 24 h using an Axio Lab.A1 microscope (Carl Zeiss AG, Oberkochen, Germany) to monitor cell density, lipid accumulation, and potential contamination. For improved visualization of lipid droplets, samples were stained with Nile red at a final concentration of 5 µg/mL prior to observation.

#### Sugar analysis

Sugars and short-chain organic acid concentrations in the fermentation media were analyzed using high-performance liquid chromatography (HPLC) with an Agilent 1260 Infinity II LC system (Agilent Technologies, Waldbronn, Germany) equipped with a diode array detector (DAD) and a refractive index detector (RID). Separation was achieved using a Rezex ROA-Organic H^+^ (8%) column (Phenomenex, Torrance, CA, USA), with 5 mM H_2_SO_4_ as the mobile phase. The analysis was performed under isocratic conditions at a flow rate of 0.5 mL/min for 40 min, with a 70 °C column oven temperature. RID detection was conducted at 40 °C [21]. Prior to analysis, all samples were centrifuged at 15,000 rcf for 10 min, and the supernatant was stored at − 20 °C. After thawing, samples were transferred to 10 kDa spin columns and filtered at 13,000 rcf and 20 °C. 197 µL of filtrate was then mixed with 3 µL of 0.5 M EDTA and subsequently analyzed via HPLC.

#### Fatty acid composition

Fatty acid composition was determined using gas chromatography with flame ionization detection (GC-FID) following fatty acid methyl ester (FAME) derivatization of lyophilized cell biomass. About 2 mg of dried biomass or an external oil standard was transferred into glass vials. Glycerol trioleate (C18:1 TAG; Sigma-Aldrich, St. Louis, MO, USA) was used as an external standard in triplicate and processed in the same way as the samples, while glyceryl trinonadecanoate (C19:0 TAG; Sigma-Aldrich, St. Louis, MO, USA) was used as an internal standard. All subsequent steps for FAME formation were performed automatically using a Multi-Purpose Sampler (MPS robotic system; Gerstel, Linthicum, MD, USA). For this, 490 μL toluene and 10 μL internal standard (10 g/L in toluene) were added to the sample and mixed for 1 min at 1000 rpm, followed by the addition of 1 mL 0.5 M sodium methoxide solution in methanol. The solution was heated to 80 °C and shaken at 750 rpm for 20 min. After cooling at 5 °C for 5 min, 1 mL of 1.25 M HCl in methanol was added, and the mixture was vortexed at 750 rpm and 80 °C for 20 min. The mixture was cooled again to 5 °C for 5 min. Subsequently, 400 μL ddH_2_O was added, and the mixture was vortexed at 1000 rpm for 30 s before 1 mL hexane was added. FAME extraction was performed by shaking three times for 12 s at 2000 rpm. The samples were centrifuged for 3 min at 1000 rpm and cooled at 5 °C before 200 μL of the organic phase was transferred to a microvial (Macherey–Nagel, Düren, Germany) for chromatography. For quantification and identification of the fatty acids, samples were analyzed by GC-FID on a GC-2025 equipped with an AOC-20i Auto Injector and AOC-20 s Auto Sampler (Shimadzu, Duisburg, Germany). Separation was performed on a Zebron ZB-WAX plus column (30 m × 0.32 mm; 0.25 μm film thickness; Phenomenex, Torrance, CA, USA). Measurements were conducted according to Engelhart-Straub et al. [22]. Retention time calibration and quantification were based on a commercial FAMEs standard mixture (Marine Oil Standard, 20 components, C14:0 to C24:1; Restek GmbH, Bad Homburg, Germany).

#### Lipid titer

Technical duplicates of each biological replicate were processed to determine the lipid titer. For this purpose, 7 mL of the unwashed fermentation broth were frozen and subsequently freeze-dried after cell disruption using a French press (EmulsiFlex-B15; AVESTIN Europe GmbH, Mannheim, Germany). Lipid extraction was performed using a modified Bligh and Dyer protocol with two extraction steps employing methanol:chloroform (2:1 v/v) [23, 24]. After complete evaporation of the chloroform phase under a nitrogen stream, the extracted lipids were quantified gravimetrically, and the respective lipid titer was calculated.

### Design of experiments

To systematically evaluate key fermentation parameters (i.e., temperature, pH, DO) that influence cell growth and lipid accumulation in oleaginous yeasts, a Box–Behnken design (BBD) was applied within a DoE framework using Design-Expert Software, Version 22.0.2 (Stat-Ease, Inc., Minneapolis, MN, USA). Each parameter was investigated at three levels within defined ranges: T (20–30 °C), pH (5.5–7.5), and DO (10–50% oxygen saturation), coded as − 1 (low), 0 (center), and + 1 (high). Detailed parameter settings and levels applied in the BBD are summarized in Table 1.

**Table 1:** Fermentation parameters and their coded and actual values used for the Box–Behnken design

Factor	Parameter	Unit	Coded level and actual value		
			Low (− 1)	Center (0)	High (+ 1)
A	T	[°C]	20	25	30
B	pH	[−]	5.5	6.5	7.5
C	DO	[%]	10	30	50

DCW [g/L], lipid titer [g/L], lipid titer (oleate) [g/L], and the proportions of saturated and unsaturated fatty acids [%] were considered as response variables. The DCW, and total lipid titer were quantified gravimetrically, while the fatty acid composition was determined by GC-FID, as described above. The lipid titer (oleate) was calculated from the quantified total lipid titer and the measured fatty acid content of oleic acid. The proportions of saturated or unsaturated fatty acids were calculated from the sum of the percentages of all saturated or unsaturated fatty acids, respectively. The required number of experimental runs *N* for the BBD was calculated using Eq. 1 [25]:

1 $$N= 2k\left(k-1\right)+{C}_{0},$$

where *k* is the number of factors and *C*_*0*_ is the number of replicates at the center point to estimate the experimental error and model curvature. To ensure statistical robustness of the model and to evaluate biological variability across fermentation runs, all experimental conditions were performed in biological duplicates. In total, 34 fermentation runs were conducted, including 10 runs at the center point (Table S1). Biological duplicates were conducted in separate 12-fold parallel bioreactor runs using independent precultures to capture biological variability between inocula.

For all predicted responses *Y*, the experimental results were fitted with a second-order polynomial function following the structure below [26]:

2 $$Y={\beta }_{0}+{\sum }_{i=1}^{k}{\beta }_{i}{x}_{i}+{\sum }_{i=1}^{k}{\beta }_{ii}{{x}_{i}}^{2}+{\sum }_{i<j}^{k}{\beta }_{ij}{x}_{i}{x}_{j},$$

where *β*_*0*_, *β*_*i*_, *β*_*ii*_, *β*_*ij*_ represent the intercept, linear, quadratic, and interaction coefficients, respectively, and *x*_*i*_ corresponds to the tested factors (T, pH, DO) with *k* = 3 as the total number of factors.

To assess the established quadratic models, statistical analysis of variance (ANOVA) was performed using Design-Expert Software, Version 22.0.2 (Stat-Ease, Inc., Minneapolis, MN, USA). The correlation coefficient (R^2^) was used to evaluate the fit of the polynomial model. The resulting models were used to identify four optimized conditions within the tested range of process parameters to maximize lipid titer, lipid titer (oleate), saturated fatty acid content, or unsaturated fatty acid content. The four predicted conditions were subsequently validated in biological triplicates in independent fermentation experiments. Additionally, five runs with the central condition (25 °C, pH 6.5, 30% DO) were included in the validation experiments for comparison (Table S3). For model validation, the mean and median deviations between the measured and predicted response variables were calculated for each of the 17 validation runs. Additionally, the percentage of validation runs within the 95% prediction interval (PI) was determined to evaluate the accuracy of the developed quadratic models.

## Results and discussion

### Experimental overview and data acquisition

**Fig. 1:**
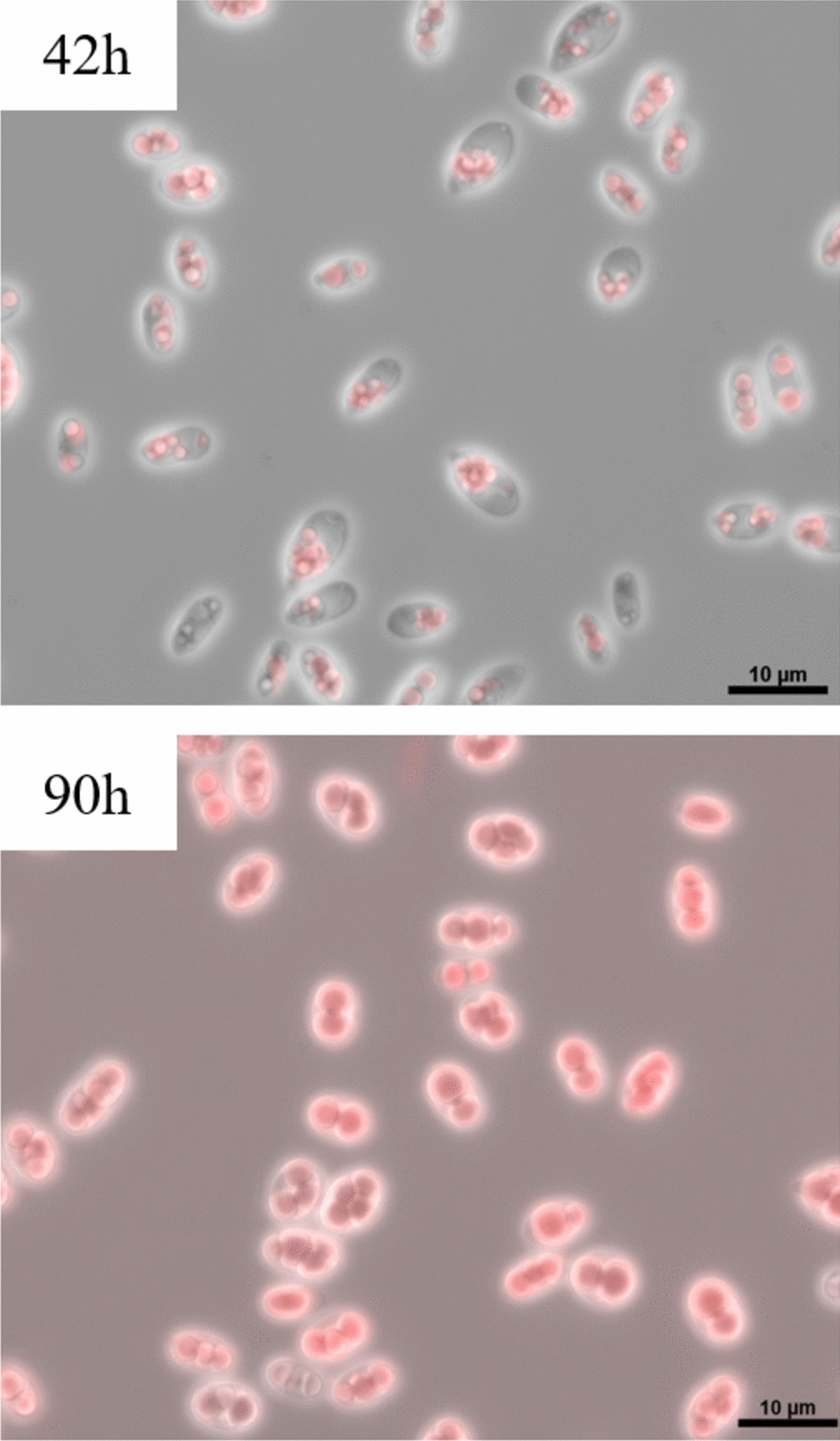
Nile red-stained *C. oleaginosus* cells after 42 h and 90 h of fermentation at 25 °C, pH 6.5, and 30% DO

To systematically evaluate the effects of temperature, pH, and DO on cell growth, lipid production, and fatty acid composition in the oleaginous yeast *C. oleaginosus*, 34 consumption-based acetic acid fermentation runs were conducted using BBD. The process parameters were tested within the following ranges: T (20–30 °C), pH (5.5–7.5), and DO (10–50%). During the fermentations, total lipid titer, lipid titer (oleate), and the proportions of saturated and unsaturated fatty acids were determined and used as response variables for model development. Sampling was performed twice a day at defined time points, while microscopic analysis, DCW, and fatty acid composition were determined every 24 h. The lipid titer was only measured at the end of the fermentation (96 h) due to limited culture volume. All tested conditions for model development, and their respective response variables after 96 h fermentation, are summarized in the supporting information Table S1. After 96 h, the measured DCW ranged from 26.5 to 48.7 g/L (0.28–0.51 g/L/h), while lipid titer varied from 18.7 to 36.2 g/L (0.19–0.38 g/L/h). These values indicate biomass and lipid productivities over time that are comparable to those reported in the literature for fed-batch cultivations of *C. oleaginosus* [20, 24]. To better visualize lipid accumulation, Fig. 1 shows Nile red-stained *C. oleaginosus* cells after 42 h and 90 h. The proportions of saturated and unsaturated fatty acids ranged from 36.1–50.4% and 49.6–63.9%, respectively, consistent with previous reports for *C. oleaginosus* under consumption-based acetic acid fermentations [20, 24, 27]. For each tested process parameter combination, the mean fatty acid profile of the biological duplicates after 96 h is shown in Figure S1. The findings indicate that increasing the temperature reduces the degree of saturation, while a lower pH increases the C18:C16 ratio.

### Model construction and quality assessment

Based on the recommendations provided by Design-Expert software, quadratic models were selected to describe each response variable. The final model structure included linear, interaction, and quadratic terms, and can generally be expressed by Eq. (2). As the assumptions for model fitting were met, data transformation was not required for all response variables (lambda = 1). For the models describing lipid titer and lipid titer (oleate), all terms involving the DO (linear, interaction, and quadratic) were statistically non-significant (*p* > 0.05) and therefore excluded from the final model, leading to the reduced equation:

3 $$Y={\beta }_{0}+{\beta }_{1}T+{\beta }_{2}pH+{\beta }_{12}TpH+{\beta }_{11}{T}^{2}+{\beta }_{22}{pH}^{2}.$$

Although the linear DO term was statistically significant (*p* < 0.05) in the models for the proportions of saturated and unsaturated fatty acids, it was intentionally excluded from the final equations in order to maintain a consistent model structure across all responses. This decision was further supported by the standardized effects, which showed that temperature and pH contributed 57% and 31%, respectively, to variations in the fatty acid composition, while DO accounted for only 12%. Model quality and predictive performance remained high despite the exclusion of the DO terms, as indicated by R^2^ values and adequate precision (see Table 2). These observations were also in alignment with previous experiments, in which no significant effect of DO on the degree of fatty acid saturation was observed at a DO range of 10–75% during cultivation at 28 °C and pH 6.5 (Figure S2). The tested DO range is consistent with values commonly applied for cultivating oleaginous yeasts, typically spanning from 5 to 80% DO [24, 28, 29]. In consumption-based acetic acid fermentations with *C. oleaginosus*, a DO level of 50% is frequently used and therefore defines the upper limit of the DO range applied in the Box–Behnken design of this study, as lower DO levels reduce the energy demand for aeration and agitation and are therefore favorable [20, 27]. The influence of the DO on *C. oleaginosus* growth has also been analyzed in the literature; however, the findings are ambiguous, reporting both positive and negative effects of elevated oxygen concentrations during cultivation [24, 28]. This highlights the difficulty of comparing DO values across different experiments, as the measured value is affected by media composition, calibration method, temperature, and bioreactor geometry [30].

**Table 2:** Fit statistics of the developed quadratic models for the response variables (*Y*)

*Y*	R^2^	Adjusted R^2^	Predicted R^2^	Adequate precision	CV [%]
Lipid titer	0.66	0.60	0.44	10.33	11.17
Lipid titer (oleate)	0.55	0.47	0.25	7.52	12.66
Fatty acids (saturated)	0.91	0.89	0.86	25.39	1.97
Fatty acids (unsaturated)	0.94	0.92	0.90	30.44	2.11

Quality and reliability of the developed quadratic models were evaluated using standard statistical indicators, as presented in Table 2. The coefficient of determination (R^2^) describes the amount of variation in the response variable explained by the model, reflecting its overall goodness-of-fit. However, as R^2^ increases with the number of terms, the adjusted R^2^ accounts for model complexity by only increasing if additional parameters improve the model more than would be expected by chance. The predicted R^2^ evaluates how well the model predicts new observations and is a key indicator of external validity [31]. A small difference (< 0.2) between adjusted and predicted R^2^ values suggests a robust model with good predictive accuracy. Adequate precision describes the signal-to-noise ratio, comparing the range of predicted values to their average standard error at the design points. A ratio greater than 4 is considered desirable [32, 33]. The coefficient of variation (CV) represents the ratio of the standard deviation to the mean and is expressed as a percentage. Lower CV values indicate higher model precision and less variability between replicates, and are generally considered favorable for model reliability [12].

The models describing fatty acid composition (saturated and unsaturated) exhibited high *R*^2^ values (0.91 and 0.94) and adjusted *R*^2^ values (0.89 and 0.92), indicating that most of the variability in the response variables is explained by the models. The slight difference between adjusted and predicted *R*^2^ values (< 0.2) further suggests good model robustness and predictive accuracy. In addition, high signal-to-noise ratios (adequate precision > 4) and low coefficients of variation (CV < 5%) support the precision and reproducibility of these models. In contrast, the lipid titer and lipid titer (oleate) models showed moderate explanatory and predictive strength, as reflected by lower *R*^2^, adjusted *R*^2^, and predicted *R*^2^ values, indicating higher experimental variability in these responses. Nevertheless, both models fulfilled the general quality criteria, with precision values above the threshold of 4 and acceptable CV values below 15%, which is considered suitable for biological systems [34]. According to Mandenius and Brundin [35], an *R*^2^ above 0.75 typically indicates a good model fit. Despite the statistical validity of the lipid titer models for trend analysis and optimization within the tested parameter range, their predictive strength should be interpreted with caution. The slightly larger difference (≈ 0.2) between adjusted and predicted *R*^2^ values further supports their limited predictive strength. The relatively high unexplained variance may result from inherent biological variability among the replicates, including cell growth or lipid accumulation differences, both of which directly affect the lipid titer. Therefore, all models are statistically acceptable; however, only the models describing the fatty acid composition should be considered highly robust and reliable for predictive purposes. The models for saturated and unsaturated fatty acid content thereby represent the first theoretical framework for *C. oleaginosus* to predict the degree of fatty acid saturation based on cultivation temperature and pH.

The final regression equations used for the response variable prediction are summarized in the supporting information Table S2. These equations include linear, interaction, and quadratic terms for the temperature and pH, and serve as the basis for further model-based optimization. For each of the 34 tested conditions for BBD development, predicted values were calculated using the final equations. To assess the predictive performance and detect potential bias, predicted values were plotted against the corresponding experimentally observed values (OP plots; observed values on the ordinate, predicted values on the abscissa), and linear regressions were performed (Supporting Information Figure S3). The respective regression lines and *R*^2^ values provide an additional measure of model quality.

Due to the identical means of predicted and observed data, as ensured by the Design-Expert model fitting, all OP regressions yielded a slope of 1 and an intercept close to 0. This behavior aligns with theoretical expectations described by Piñeiro et al. [36] and further supports the accuracy and symmetry of the developed models, confirming the absence of systematic prediction bias.

### Model interpretation and influence of process parameters

**Fig. 2:**
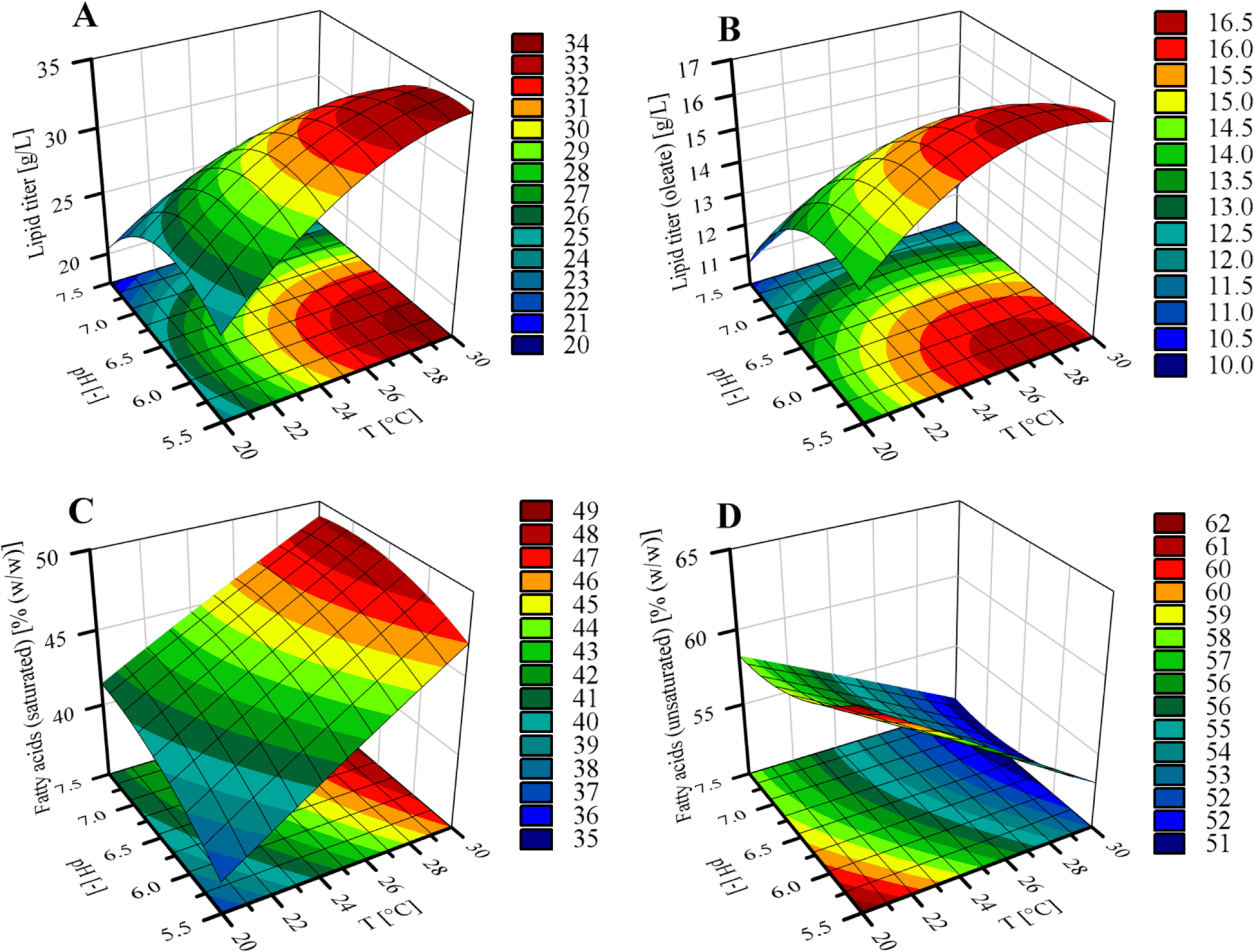
Response surface plots showing the influence of process parameters on response variables. The combined effects of temperature (T) and pH are shown in **A** total lipid titer, **B** lipid titer (oleate), **C** saturated fatty acids, and **D** unsaturated fatty acids. Color gradients indicate the value of the response variables across the experimental range

To gain a deeper understanding of the individual and interactive effects of process parameters on the response variables, the response surface plots (Fig. 2) and the corresponding coded equations of the quadratic models (Table 3) were evaluated.

**Table 3:** Coded factors of quadratic response surface models

*Y*	*β*_*0*_	*β*_*1*_	*β*_*2*_	*β*_*12*_	*β*_*11*_	*β*_*22*_
Lipid titer [g/L]	31.20	3.46	− 3.47	− 1.64	− 2.24	− 3.29
Lipid titer (oleate) [g/L]	15.61	0.76	− 2.12	− 0.51	− 1.16	− 1.37
Fatty acids (saturated) [% (w/w)]	44.75	4.37	1.61	− 0.58	− 0.14	− 0.96
Fatty acids (unsaturated) [% (w/w)]	55.18	-4.21	− 1.61	0.58	− 0.03	1.12

Predicted responses *Y* are described by the equation: $Y={\beta }_{0}+{\beta }_{1}{X}_{1}+{\beta }_{2}{X}_{2}+{\beta }_{12}{X}_{1}{X}_{2}+{\beta }_{11}{{X}_{1}}^{2}+{\beta }_{22}{{X}_{2}}^{2}$, where X_1_ and X_2_ represent the coded values of temperature and pH, respectively

#### Total and oleate lipid titer

The total lipid titer is a key response variable that reflects the quantitative outcome of microbial oil production, and is therefore an important target for process optimization. Variations in process parameters can directly affect the growth of the oleaginous yeasts, or their capacity to accumulate intracellular lipids, both influencing the final lipid titer and overall lipid productivity [11]. Lipid productivity thereby indicates the most cost-impacting factor in biodiesel production, as described by Kumar et al. [37]. In addition to the total lipid titer, the oleate lipid titer was included as a relevant response variable due to its specific industrial relevance. Oleic acid offers favorable fuel properties for biodiesel applications, including a desirable melting point, high oxidative stability, suitable kinematic viscosity, and a high heat of combustion [38]. In addition, oleic acid can be enzymatically converted into 10-(R)-hydroxy stearic acid (10-HSA), a fatty acid-derived fine chemical used in the lubricant and cosmetic industries [18]. Since oleate (C18:1) typically represents the predominant fatty acid produced by *C. oleaginosus*, similar effects of the process parameters on total lipid and oleate titers can be expected.

As demonstrated in the response surface plots, temperature and pH exhibited similar effects on both lipid titer (Fig. 2A) and oleate titer (Fig. 2B). In general, lipid titer increased with rising temperatures or decreasing pH values, reaching maximum values at approximately 28–30 °C and pH levels around 5.5–6.0. In contrast, low temperatures (20 °C) or alkaline conditions (pH 7.5) markedly reduced lipid accumulation, regardless of the respective pH or temperature setting. The response surface plot for oleate lipid titer displayed a similar pattern, suggesting a comparable dependency on the process parameters. However, the temperature optimum was shifted slightly toward lower values (26–30 °C), which is consistent with literature reports, indicating that lower temperatures promote the synthesis of unsaturated fatty acids, such as oleic acid in oleaginous yeasts [8, 16]. This trend can be explained by temperature-dependent regulation of desaturase enzymes, which introduce double bonds into fatty acid chains. The thereby increased degree of unsaturation enhances membrane fluidity, counteracting the increased membrane rigidity observed at lower cultivation temperatures [39]. The coded regression coefficients in Table 3 support the observed trends in the response surface plots. In these coded equations, the values for temperature and pH are normalized to a range from -1 to 1, enabling direct comparison of the relative influence of each factor. For total lipid titer, temperature and pH showed antagonistic linear effects (β_1_ = 3.46; β_2_ = − 3.47) with a similar strength. However, the larger quadratic term for pH (β_22_ = − 3.29 vs. β_11_ = − 2.24) indicates a more pronounced curvature. For the oleate titer, linear and quadratic coefficients for the pH value were higher than those for temperature. The observations therefore suggest a greater overall influence of pH on total lipid titer, and lipid titer of oleate within the tested range. This stronger effect of pH can likely be attributed to its direct impact on acetic acid uptake, the primary carbon source utilized for lipid production in this study. In yeast cells, acetic acid can be taken up either as acetate via active transporters or as undissociated acetic acid via passive diffusion [40]. Lower pH values increase the fraction of undissociated acetic acid (15.4% at pH 5.5 and 1.8% at pH 6.5), thereby enhancing substrate availability and supporting higher lipid production. Studies from Duman‑Özdamar et al. and Cui et al. also analyzed the influence of process parameters on the lipid production by *C. oleaginosus* using response surface methodology [8, 17]. In shake flask experiments conducted under nitrogen-limited conditions with glycerol as the carbon source, both studies identified an optimal temperature for lipid production around 30 °C. Cui et al. further determined a pH optimum of 6.0 for lipid accumulation [17]. In the present study, a similar temperature optimum was observed, while the pH optimum was slightly lower (5.7), which may be explained by the pH-based feeding strategy applied in the bioreactor setup.

#### Saturated and unsaturated fatty acid composition

As the degree of saturation of the yeast oil produced determines its physicochemical properties, changes in the fatty acid profile can enable a wide range of end-use applications. Therefore, the influence of temperature and pH on the fatty acid composition was analyzed using the respective response surface plots (Fig. 2C, D) and the coded regression equations (Table 3). In general, an inverse relationship between the proportions of saturated and unsaturated fatty acids was observed. A strong positive correlation was found between the degree of saturation and the cultivation temperature. This effect appeared linear within the tested parameter range (20 °C ≤ T ≥ 30 °C), as indicated by the response surface plots and the coded factors (see Fig. 2C, D, and Table 3). The influence of the pH on the degree of saturation was less pronounced. However, higher pH values tend to increase saturation. Cultivation at elevated temperature (30 °C) and high pH (pH 7.5) resulted in a higher proportion of saturated fatty acids, while the opposite process parameters (*T* = 20 °C, pH 5.5) reduced saturation.

The observed influence of temperature on the fatty acid profile aligns with previous literature, which also reports an increase in saturation at elevated temperatures for *C. oleaginosus* and other oleaginous yeasts [8, 16, 41]. While pH is known to significantly affect cell growth and, consequently, lipid titer in oleaginous yeasts (see 3.2.1), its direct impact on the fatty acid profile is generally considered negligible [15, 42, 43]. In contrast, the observations from this study suggest that during consumption-based acetic acid fermentation, higher pH values promote fatty acid saturation in *C. oleaginosus*. This pH influence on the degree of saturation can be explained by differences in substrate availability during fermentation at varying pH levels. As described above, lower pH levels enhance the uptake of undissociated acetic acid via passive diffusion, which is consistent with the observed differences in overall acetic acid consumption at 25 °C. The total amount of acetic acid consumed during the 96 h cultivation increased with decreasing pH (pH 5.5: 1.69 ± 0.13 g/L/h, *n* = 3; pH 6.5: 1.36 ± 0.19 g/L/h, *n* = 14; pH 7.5: 1.14 ± 0.36 g/L/h, *n* = 3). Consequently, lower pH values increase the intracellular availability of acetic acid, which in turn affects the metabolic fluxes involved in lipid biosynthesis and thereby alters the degree of fatty acid saturation. Thus, pH most likely affects the lipid profile only indirectly, by modulating acetic acid availability rather than directly influencing lipid metabolism. Similar substrate-induced shifts in fatty acid profiles have been reported for other oleaginous yeasts [9–11]. Specifically, acetic acid as a carbon source has been shown to slightly favor the formation of more unsaturated fatty acids compared to other organic acids [44]. Previous shake flask experiments with *C. oleaginosus* in nitrogen-limited batch conditions showed no notable effect of pH on the fatty acid saturation (data not shown), thereby supporting the hypothesis of a substrate-dependent shift during consumption-based acetic acid fermentations.

### Model validation under optimized conditions

To validate the predictive performance of the developed quadratic models, optimization criteria were defined for each response variable. The optimal combinations of process parameters within the tested ranges were identified by the models to maximize each target response and were selected for validation experiments (see Table 4). Since the developed models used for parameter prediction did not include a DO term, the oxygen saturation during fermentation was set to 10% in all cases to reduce operating costs by minimizing the required agitation and aeration [28, 45]. The results thereby indicate that the growth and lipid production of *C. oleaginosus* are largely unaffected by low dissolved oxygen levels (10%). This provides valuable guidance, especially for process scale-up, as cultivation at reduced DO lowers energy requirements and consequently reduces overall production costs. These optimized conditions were tested in biological triplicates together with five further fermentation runs under central conditions (25 °C, pH 6.5, 30% DO), to further take biological variability into account.

**Table 4:** Optimized process parameter settings for maximizing each response variable *Y*_*opt*_

*Y*_*opt*_	T [°C]	pH [−]	DO [%]
Central condition	25.0	6.5	30
Lipid titer	30.0	5.7	10
Lipid titer (oleate)	27.6	5.6	10
Fatty acids (saturated)	30.0	7.0	10
Fatty acids (unsaturated)	20.0	5.5	10

All measured data for the respective response variables after 96 h of cultivation are summarized in the supporting information Table S3. The predictive performance of the developed quadratic models was evaluated based on these 17 validation experiments. Therefore, the mean and median deviations between the measured and predicted response variables, as well as the percentage of validation runs within the 95% prediction interval were calculated (see Table S4).

For total lipid titer and lipid titer (oleate), which had moderate R^2^ values of 0.66 and 0.55, respectively, the mean deviations between predicted and observed values were slightly above 10% (12.9% and 14.0%). Nevertheless, 88.2% and 94.1% of the 17 validation runs fell within the 95% PI, indicating an acceptable model reliability despite the limited goodness-of-fit. The larger deviations are likely to be attributed to the biological variability between fermentations. As the 95% prediction intervals are much wider than the 95% confidence intervals, both models can support general trend identification and process understanding. However, for precise optimization of the respective response variables, further refinement of the models is required. The quadratic models describing the fatty acid composition of the yeast oil showed comparable model reliability based on the 95% PI coverage (88.2% for saturated and 94.1% for unsaturated fatty acids). However, the mean deviation between predicted and observed values was much lower (2.4% and 2.1%, respectively), reflecting the superior model fit as indicated by the high *R*^2^ values (*R*^2^_saturated_ = 0.91; *R*^2^_unsaturated_ = 0.94). These results confirm that the models for fatty acid distribution not only describe the experimental trends well, but can also provide accurate predictions of the fatty acid composition within the tested parameter range. Therefore, the required process conditions for obtaining tailored fatty acid profiles can be reliably predicted, enabling different end-use applications of the yeast oil by simply adjusting temperature and pH during fermentation.

**Fig. 3:**
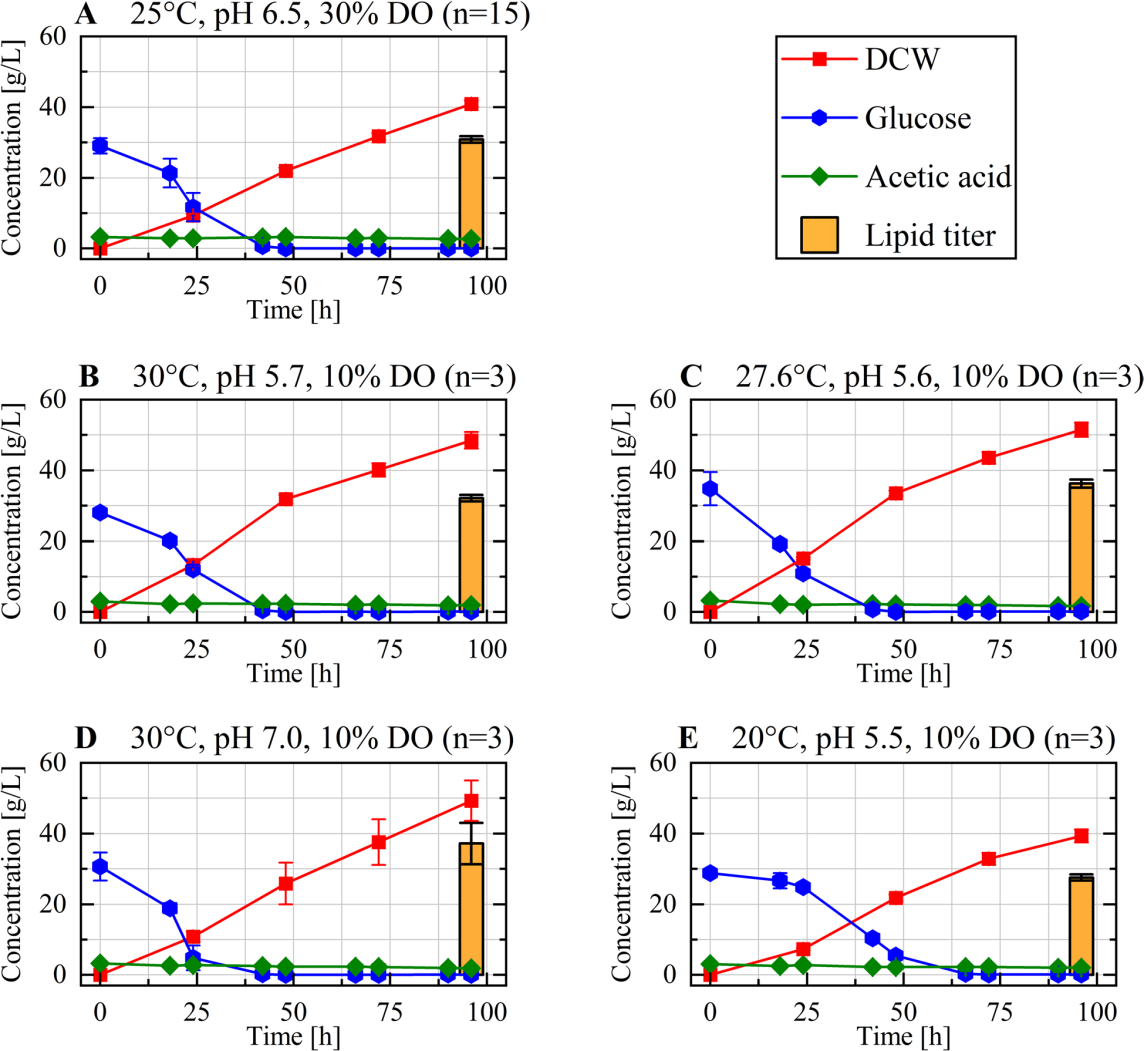
Biomass formation, substrate utilization, and lipid production during the validation experiments. The development of these cultivation parameters is shown for the central condition (**A**) and the optimized conditions for total lipid titer (**B**), lipid titer (oleate) (**C**), saturated fatty acid content (**D**), and unsaturated fatty acid content (**E**). Values represent mean ± standard deviation of *n* biological replicates (*n* = 15 for the central condition; *n* = 3 for each optimized condition)

After confirming the predictive reliability of the developed models, the fermentation experiments conducted under the respective optimized and central conditions were analyzed in more detail to evaluate biomass formation, substrate utilization, and product accumulation. Mean values and standard deviations for DCW, glucose, and acetic acid concentrations, as well as the lipid titer, are presented in Fig. 3 for each validation condition. In all conditions, the initial glucose concentration of 30 g/L was depleted within the first 48 h, except for the condition at 20 °C and pH 5.5 (Fig. 3E), where 5.5 g/L glucose remained at that time point. These observations are consistent with the glucose consumption patterns observed both during the fermentation runs used for model development and in fermentations reported by Rerop et al. under similar conditions with 30 g/L initial glucose at 28 °C and pH 6.5 [20]. In fermentations conducted at 20 °C, residual glucose was frequently detected after 48 h, whereas at 25 °C or 30 °C, complete glucose depletion typically occurred within this time frame, regardless of pH and DO concentration (data not shown). This observation can be attributed to lower growth rates *μ* at 20 °C during the first 24 h, which result in slower glucose uptake compared to 25 °C and 30 °C (at pH 6.5: μ_20°C_ = 0.069 ± 0.004 1/h, *n* = 4; μ_25°C_ = 0.088 ± 0.008 1/h, *n* = 15; μ_30°C_ = 0.089 ± 0.007 1/h, *n* = 4). The lower growth rates at 20 °C, also explain the reduced acetic acid consumption rate at this temperature. Acetic acid concentrations remained stable between 1.5 and 3.5 g/L throughout the cultivation across all tested conditions. This underscores the effectiveness of the pH-based acetic acid feeding strategy developed by Masri et al., which enables balanced substrate availability and avoids both substrate limitation and accumulation. This approach adapts dynamically to the actual substrate uptake rate, regardless of the biological performance of the culture [27].

Biomass increased almost linearly in all conditions without signs of saturation or limitation, indicating that prolonged cultivation would likely result in higher final DCW values. This observation is consistent with findings by Masri et al. [27], who reported continuous biomass growth over a period of 120 h of fermentation. After 96 h, the highest overall DCW (51.5 g/L) was observed under the optimized conditions for oleate lipid titer at 27.6 °C and pH 5.6 (Fig. 3C). In contrast, the lowest overall DCW (39.3 g/L) was measured at the lowest tested temperature (20 °C, pH 5.5), representing the optimized conditions for maximizing unsaturated fatty acid production (Fig. 3E). As expected, the growth of *C. oleaginosus* was reduced at lower temperatures, in agreement with previous studies [8, 17]. However, the biomass concentration obtained at the central conditions (25 °C, pH 6.5) was only slightly higher (40.9 g/L after 96 h), highlighting the robustness of the strain across a moderate temperature range. The highest lipid titer after 96 h was observed under the conditions maximizing the oleate lipid titer (36.3 g/L at 27.6 °C, pH 5.6; Fig. 3C), and for saturated fatty acid production (37.2 g/L at 30 °C, pH 7.0; Fig. 3D), whereas the lowest titer (27.6 g/L) was measured under the optimized condition for unsaturated fatty acid accumulation (20 °C, pH 5.5; Fig. 3E). The overall lipid content varied between 65 and 75% (w_lipid_/w_DCW_) across all tested conditions, indicating that differences in lipid titer primarily result from variations in biomass formation rather than from a reduced capacity for lipid accumulation. For a better economic evaluation, the product yield (substrate-to-product conversion ratio) was calculated for the respective optimized conditions. The highest product yield was observed under the condition optimized for saturated fatty acid production, with 0.24 ± 0.01 g/g, while the center condition reached 0.23 ± 0.02 g/g. The lowest yield was obtained under the condition optimized for total lipid titer (0.20 ± 0.01 g/g), likely due to the increased acetic acid consumption observed at lower pH values. Overall, the product yields determined in this study are comparable to values recently reported in the literature for consumption-based acetic acid fermentations with *C. oleaginosus* [20]. To enable better comparison with previous studies on consumption-based acetic acid fermentations with *C. oleaginosus*, the lipid productivity (amount lipid produced per unit volume and time) was calculated. In line with the lipid titer data, the highest productivities were observed under the optimized conditions for oleate lipid titer (27.6 °C, pH 5.6), and saturated fatty acid production (30 °C, pH 7.0), with 0.38 g/L/h and 0.39 g/L/h, respectively. Under the central condition (25 °C, pH 6.5), a lipid productivity of 0.32 g/L/h was achieved, while the lowest productivity was observed at 20 °C, pH 5.5 (0.29 g/L/h). Using comparable feeding setups and media compositions with 30 g/L initial glucose, Rerop et al. and Stellner et al. reported lipid titers of 18.5 g/L and 16.8 g/L after 72 h at 28 °C and pH 6.5, with a lipid content of 53% (w_lipid_/w_DCW_). These values correspond to lipid productivities of 0.26 g/L/h and 0.23 g/L/h, respectively [20, 24]. This demonstrates that lipid production in *C. oleaginosus* can be substantially enhanced through a DoE-based optimization approach by adjusting key process parameters, such as temperature and pH, to promote both cell growth and lipid accumulation. This resulted in up to 16% higher lipid productivity compared to the central conditions and up to 50% higher compared to previously reported values in the literature [20]. While the R^2^ value of the oleate lipid titer model indicates only moderate predictive power (*R*^2^ = 0.55), the observed increases in lipid productivity relative to the center condition nevertheless suggest a positive effect of the optimized process parameters on lipid accumulation in *C. oleaginosus,* thereby providing an important indicative understanding of the process behavior.

As this study was conducted using only glucose and acetic acid as carbon sources, further increases in lipid production may be achieved by combining the process optimization strategies presented here with alternative carbon sources or feeding strategies, as well as by increasing the starting sugar concentration, as explored in other studies. One such approach involves the use of second-generation feedstocks, such as paper mill sludge, macro- and microalgal biomass, or corn stover hydrolysate, which are considered low-cost and sustainable substrates for microbial lipid production [20, 46–48]. For example, Rerop et al. demonstrated that the use of a lignocellulosic hydrolysate derived from the pulp and paper industry significantly enhanced both biomass formation and lipid titer compared to a glucose-based control [20].

**Fig. 4:**
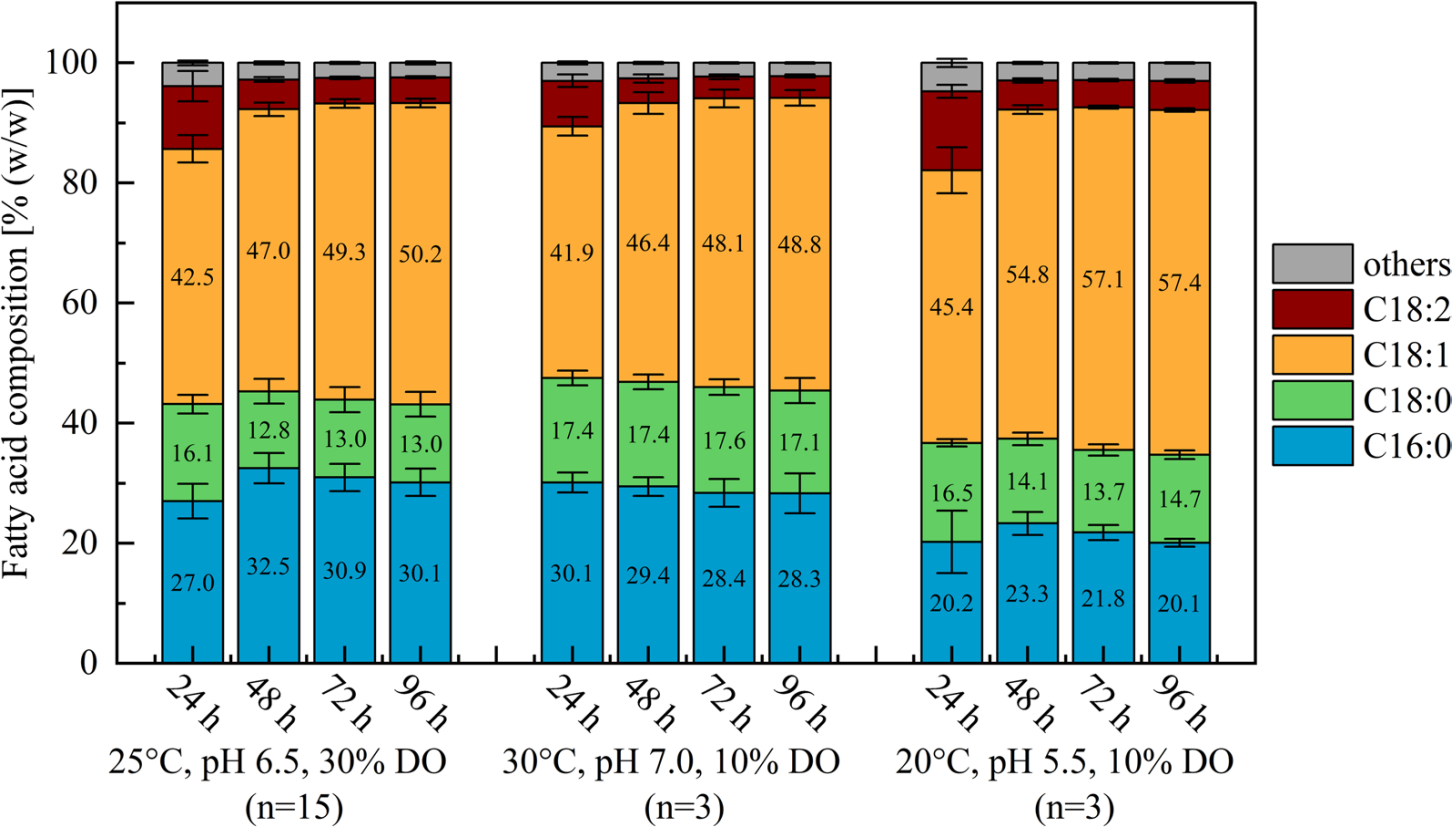
Temporal development of the fatty acid profile of *C. oleaginosus* during consumption-based acetic acid fermentation under validation conditions. Shown are changes in fatty acid composition between 24 and 96 h for the central condition (25 °C, pH 6.5, 30% DO) and two optimized conditions: for saturated fatty acid content (30 °C, pH 7.0, 10% DO), and for unsaturated fatty acid content (20 °C, pH 5.5, 10% DO). Values represent mean ± standard deviation of *n* biological replicates

To better understand how process parameters such as temperature and pH influence the fatty acid profile over time, the composition of fatty acids was analyzed every 24 h. Figure 4 shows the temporal development of the fatty acid composition of *C. oleaginosus* under the central conditions (25 °C, pH 6.5, 30% DO) as well as under the optimized conditions for saturated fatty acid content (30 °C, pH 7.0, 10% DO), and unsaturated fatty acid content (20 °C, pH 5.5, 10% DO). Comparing the three conditions, differences in fatty acid composition were already observed after 24 h, indicating a rapid adaptation of lipid biosynthesis to the specific temperature and pH conditions. Only minor changes were observed after 48 h, and from 72 h onwards, the fatty acid profiles remained largely stable until 96 h. These trends were consistent across the other validation fermentations as well as the BBD-based experimental runs (data not shown). The results suggest that temperature and pH have a rapid effect on the fatty acid composition and are therefore also relevant for shorter fermentation processes.

**Fig. 5:**
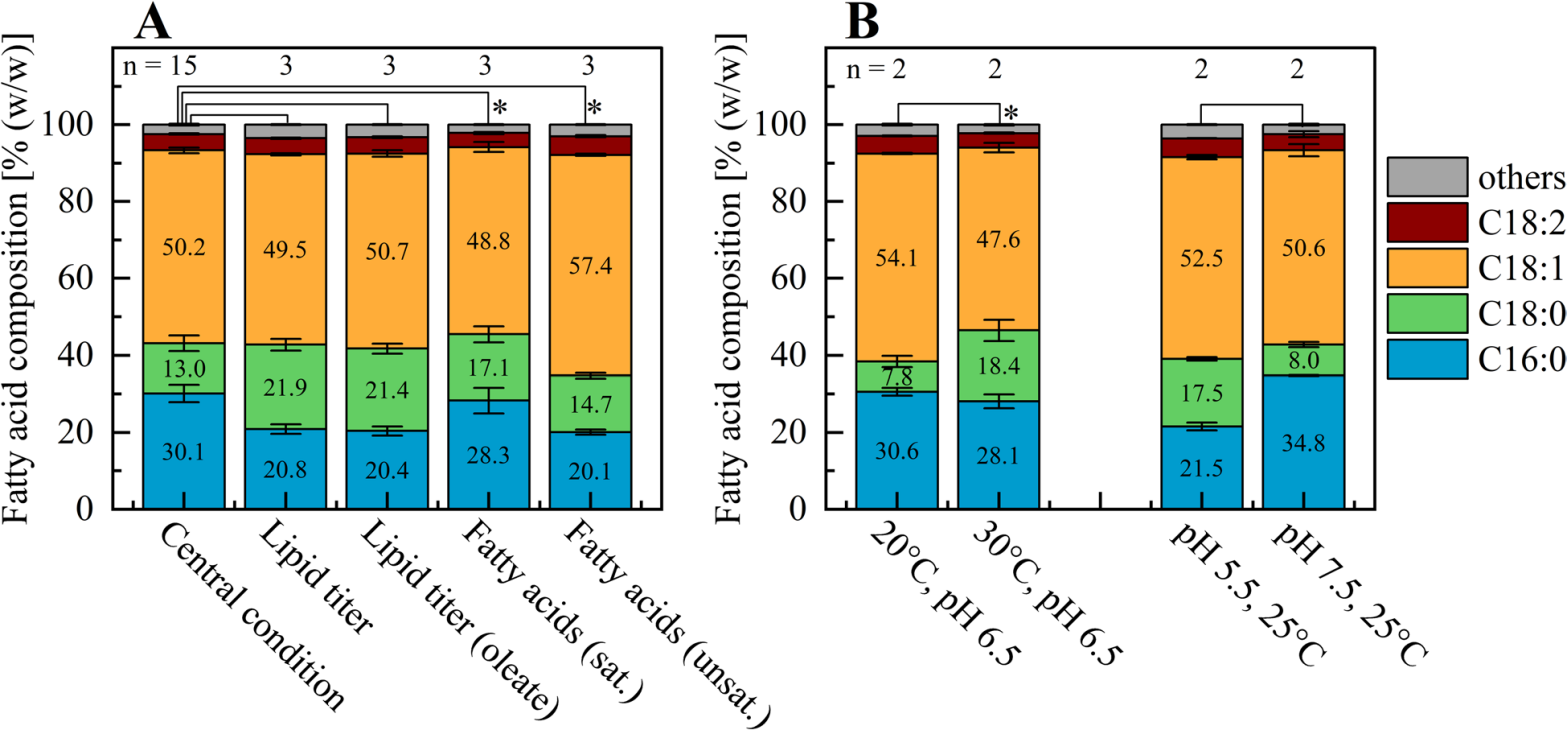
Fatty acid profiles of *C. oleaginosus* after 96 h fermentation under varying process parameters. **A** Shows the fatty acid composition under the central conditions (25 °C, pH 6.5, 30% DO) and four optimized conditions: for total lipid titer (30 °C, pH 5.7, 10% DO), lipid titer (oleate) (27.6 °C, pH 5.6, 10% DO), saturated fatty acid content (30 °C, pH 7.0, 10% DO), and unsaturated fatty acid content (20 °C, pH 5.5, 10% DO). In **B**, the impact of temperature (left) and pH (right) on the fatty acid profile under conditions used for BBD development is shown. The DO level was set to 10% saturation in these cases. Values represent mean ± standard deviation of *n* biological replicates in all cases. Statistically significant differences in the overall degree of fatty acid saturation between groups are indicated with * (*p* < 0.05)

As the optimized conditions for saturated and unsaturated fatty acid content were specifically selected to represent the maximum achievable shifts in fatty acid composition within the tested parameter range, the corresponding fatty acid profiles from the validation experiments are shown in Fig. 5A. The central conditions (25 °C, pH 6.5), as well as the optimized conditions for total (30 °C, pH 5.7) and oleate lipid titer (27.6 °C, pH 5.6), resulted in a similar degree of saturation of approximately 44%. This comparable level of saturation aligns well with the trends observed during model development (Fig. 2C, D), where temperature and pH exhibited antagonistic effects on the degree of saturation. While the elevated temperature in the optimized lipid titer condition increased saturation, the lower pH appeared to reduce it, resulting in a balanced overall effect. Interestingly, despite the similar total saturation, the distribution between palmitic acid (C16:0) and stearic acid (C18:0) varied across the three conditions. In the fermentations optimized for saturated (30 °C, pH 7.0) and unsaturated fatty acid composition (20 °C, pH 5.5), the synergistic effects of temperature and pH became more evident. Saturation degrees of 47% and 36.6% were observed, respectively. These results demonstrate that the adjustment of process parameters such as temperature and pH alone can induce an absolute shift in saturation of more than 10%.

Shaigani et al. developed genetically engineered *C. oleaginosus* strains using the CRISPR/Cas9 system. In shake flask cultivations under nitrogen-limited conditions, overexpression of ∆9-fatty acid desaturase genes (D9FAD) led to an increase in oleic acid and a decrease in total saturated fatty acids by approximately 3.3% after 96 h, compared to the wild-type strain. When alternative promoter systems such as TEF1 or AKR1 were used to control D9FAD expression, the saturation level could be modulated more significantly, resulting in an increase in saturation of up to 20% [49]. These findings highlight the potential of metabolic engineering to tailor fatty acid profiles in oleaginous yeasts. However, the genetic modification of oleaginous yeasts is still technically challenging, and concerns regarding strain stability and regulatory hurdles, particularly in food and feed applications, can limit its industrial application [50]. For applications where the use of genetically modified organisms is permitted, further adaptation of the fatty acid profile could be achieved through a combination of targeted strain engineering and precise adjustment of cultivation parameters.

To better understand and visualize the influence of process parameters on fatty acid composition, Fig. 5B shows data from the fermentation runs used for model development, illustrating the respective effects of temperature and pH while keeping all other conditions constant. As previously discussed, increasing the temperature from 20 °C to 30 °C leads to a higher degree of saturation. At a constant pH of 6.5 and 10% DO, a temperature increase of 10 °C resulted in an 8.5% enhancement in saturation. These findings are consistent with the results from validation experiments, where simultaneous changes in temperature and pH led to more pronounced changes in the fatty acid profile. A likely explanation for the temperature dependence of fatty acid saturation is the adaptive response of yeast cells to lower temperatures by upregulating the expression of fatty acid desaturases, as previously reported for *Yarrowia lipolytica* [51]. The distribution between palmitic acid and stearic acid appears to be only slightly affected by temperature, with an increase of 10 °C leading to a change in palmitic acid content of only 2.5%.

An increase in pH from 5.5 to 7.5, while maintaining 25 °C and 10% DO, resulted in a slight increase in overall fatty acid saturation of 2.4%. However, a lower pH reduced the content of palmitic acid (C16:0) and increased the stearic acid (C18:0) content. A reduction in pH from 7.5 to 5.5 led to a 13.3% decrease in C16:0 content. This pH-dependent shift in the C16/C18 ratio was also consistently observed in the other validation fermentations.

A possible explanation could be the enhanced uptake of acetic acid under more acidic conditions, as previously discussed. In yeasts, the elongation of palmitic acid to stearic acid occurs in the endoplasmic reticulum and is catalyzed by the *Elo2* fatty acid elongase [19]. This reaction uses malonyl-CoA as a two-carbon donor and NADPH as a reducing agent [52]. Increased acetic acid availability at low pH may elevate intracellular acetyl-CoA levels, leading to higher malonyl-CoA concentrations and consequently enhanced elongation of C16:0 to C18:0 [53, 54]. Consequently, the fatty acid profile of the produced yeast oil can be tailored by adjusting cultivation parameters, with temperature primarily influencing the degree of saturation, and pH predominantly affecting the C16/C18 ratio.

### Industrial application and product customization through process control

To assess the industrial applicability of the produced yeast oil as a biofuel, key fuel-related parameters such as cetane number, iodine value, higher heating value, and kinematic viscosity were calculated based on the respective fatty acid composition using equations provided by Sergeeva et al. [55]. As shown in Table 5, all produced yeast oils, regardless of the cultivation conditions, fulfilled the threshold values of the European biodiesel standard and thus represent suitable alternatives to palm oil. In particular, the oil produced under conditions optimized for saturated fatty acid content (30 °C, pH 7.0, 10% DO) exhibited the highest cetane number, indicating higher combustibility of the fuel, and the lowest iodine number, indicating a higher oxidative stability of the fuel. These properties are both favorable for biofuel production. Compared to conventional vegetable oils, such as palm, rapeseed, soybean, and high-oleic sunflower oil (HOSO), the yeast-derived oils showed comparable or even superior fuel properties, particularly in terms of cetane number and oxidative stability. In addition to biodiesel, yeast oil may also represent a promising feedstock for sustainable aviation fuels. A high proportion of saturated fatty acids is favorable in this context, as unsaturated fatty acids require an additional hydrogenation step to convert double bonds into saturated hydrocarbons in the HEFA (Hydroprocessed Esters and Fatty Acids) pathway [56].

**Table 5:** Degree of saturation and estimated fuel properties of yeast and plant-derived oils. Fuel-related parameters of the different yeast oils obtained in this study were calculated based on the respective measured fatty acid profiles

Biodiesel source	SFAs [%_w/w_]	UFAs [%_w/w_]	Cetane number [−]	Iodine value [−]	Higher heating value [MJ/kg]	Kinematic viscosity [mm^2^/s]
Yeast oil^a^						
Central conditions	44.7	55.3	63.3	53.9	39.5	4.1
Fatty acids (sat.)	47.0	53.0	64.0	51.5	39.5	4.1
Fatty acids (unsat.)	36.6	63.4	62.3	62.6	39.6	4.1
Vegetable oil^b^						
Palm oil	48.0	52	61.4	56.4	39.4	3.9
Rapeseed oil _(Canola)_	8.0	92.0	51.5	115.1	39.5	3.8
Sunflower oil _(HOSO)_	9.8	90.2	57.6	88.8	39.6	4.1
Soybean oil	14.9	85.1	45.5	139.4	39.4	3.6
Biodiesel (standards)^c^						
EN14213	–	–	–	–	≥ 35	–
EN14214	–	–	≥ 51.0	≤ 120	–	3.5–5.0

^a^ Fatty acid profiles of the yeast oils (*C. oleaginosus*) after 96 h cultivation under central conditions (25 °C, pH 6.5, 30% DO), or under optimized conditions for maximum saturated fatty acid (SFA) content (30 °C, pH 7.0, 10% DO), or unsaturated fatty acid content (20 °C, pH 5.5, 10% DO)

^b^ Fatty acid profiles of the plant oils (palm oil, rapeseed oil (canola), high-oleic sunflower oil (HOSO), and soybean oil) obtained from literature [57]. Content of saturated (SFAs) and unsaturated fatty acids (UFAs), as well as derived fuel properties, were calculated using empirical equations [55]

^c^ Threshold values of the European biodiesel standard EN14213 and EN14214 are used for comparison

As monounsaturated fatty acids represent an excellent compromise between oxidative stability and favorable fluidity across a wide temperature range, oils with high oleic acid content are of particular industrial interest [58]. Potential applications for the yeast-derived oil include biolubricants, hydraulic fluids, and insulating oils for electrical transformers [58]. In this context, yeast oil offers a sustainable oleic acid source, with the potential for higher productivities and significantly reduced land requirements compared to conventional vegetable-based feedstocks. Prem et al. observed that oil derived from *C. oleaginosus* enabled the most efficient bioconversion of oleic acid into the fine chemical 10-HSA, outperforming vegetable oils such as HOSO and rapeseed oil [18]. Moreover, the oleic acid content of yeast oil can be selectively increased by adjusting the cultivation parameters. A lower temperature and pH (20 °C, pH 5.5) enhanced the C18:1 content by nearly 10%, reaching 57.4% of total fatty acids, compared to cultivation at 30 °C and pH 7.5 (48.8% (w/w) C18:1).

As a change of cultivation parameters showed the possibility to adjust both the saturation and the C16/C18 ratio of the produced yeast oil without the need for genetic engineering, the results are of particular interest for the food industry. This is especially relevant for the production of cocoa butter equivalents (CBEs), where a defined ratio of palmitic, stearic, and oleic acid is essential to replicate the physical and sensory properties of natural cocoa butter, such as melting characteristics and texture [59]. Cacao butter typically exhibits the following fatty acid composition: 21.7–27.0% C16:0, 30.6–39.2% C18:0, 29.4–35.4% C18:1, 2.2–3.4% C18:2, < 2.0% C20:0 [60]. Cultivation of *C. oleaginosus* at 30 °C and a pH around 6.5 was found to enhance the degree of saturation while maintaining the palmitic acid content within the target range. Through additional optimization of the cultivation medium, the lipid profile can be further tailored to match that of cocoa butter, without the need for genetically modified organisms [9–11]. Moreover, this biotechnological approach offers key advantages over conventional plant oil-based CBEs, as it reduces dependency on agricultural land and climate change effects. Further, it enables more sustainable production with significantly lower resource requirements [61].

## Conclusion

This study aimed to improve lipid production in the oleaginous yeast *C. oleaginosus* ATCC 20509 and modify its fatty acid profile without the need for genetic engineering. A three-level, three-factor Box–Behnken design was employed to evaluate the influence of temperature, pH, and dissolved oxygen concentration in the fermentation medium on lipid titer, oleate lipid titer, and the proportions of saturated and unsaturated fatty acids produced by the oleaginous yeast. Statistical analysis revealed that only temperature and pH significantly affected the response variables. Using the developed quadratic models and the predicted optimal process parameters, lipid productivity could be increased by up to 50% compared to literature values. Adjusting only temperature and pH also enabled targeted modification of the lipid fatty acid composition. The degree of saturation could be adjusted within a range of more than 10%, with temperature being the main influencing factor, while pH allowed for modulation of the C16/C18 ratio, leading to a 13% change in palmitic acid content. These results highlight the potential of a DoE approach to systematically identify the influence of cultivation parameters for process modification and optimization. Cultivation conditions were thereby identified as an important parameter for adjusting both the degree of saturation and the C16/C18 ratio, which complements the known effects of media composition on the fatty acid profile. Combining these strategies allows tailoring of *C. oleaginosus'* lipid composition for various applications such as biofuels, biolubricants, or cocoa butter equivalents, without resorting to genetic engineering. These insights support the transition toward a circular and bio-based economy, enabling an efficient production of customized microbial oils as renewable alternatives to conventional plant-based lipids.

## Supplementary Information

Supplementary Material 1.

## Data Availability

Data used for model development and validation are included within the manuscript and supplementary information files. Additional raw datasets (e.g., time-resolved DCW and lipid profiles) are available from the corresponding author upon request.

## Abbreviations

ANOVA: Analysis of variance
BBD: Box–Behnken design
CBEs: Cocoa butter equivalents
CV: Coefficient of variation
DAD: Diode array detector
DCW: Dry cell weight
DO: Dissolved oxygen concentration
DoE: Design of experiments
D9FAD: ∆9-Fatty acid desaturase genes
FAME: Fatty acid methyl ester
GC-FID: Gas chromatography with flame ionization detection
HEFA: Hydroprocessed esters and fatty acids
HOSO: High-oleic sunflower oil
HPLC: High-performance liquid chromatography
OD_600_: Optical density at 600 nm
OP: Plots, observed values on the ordinate, predicted values on the abscissa
PI: Prediction interval
RID: Refractive index detector
RSM: Response surface methodology
SCO: Single-cell oil
SFAs: Saturated fatty acids
T: Temperature
TAG: Triacyl glyceride
UFAs: Unsaturated fatty acids
10-HSA: 10-(R)-Hydroxy stearic acid
