# Supplementary material for: Influence of process parameters on single-cell oil production by Cutaneotrichosporon oleaginosus using response surface methodology
Source: Biotechnol Biofuels Bioprod. 2025 Nov 19;18:115. doi: 10.1186/s13068-025-02717-3 (PMC12628619; doi:10.1186/s13068-025-02717-3)
Supplement: Supplementary file 1 — Supplementary Material 1. [file 13068_2025_2717_MOESM1_ESM.docx]

Research Paper

Influence of process parameters on single-cell oil production by *Cutaneotrichosporon oleaginosus* using response surface methodology

Max Schneider^1^, Felix Melcher^1^, Robert Fimmen^1^, Johannes Mertens^1^, Daniel Garbe^1^, Michael Paper^1^, Marion Ringel^1^, and Thomas Brück^1*^

^1^ Werner Siemens-Chair of Synthetic Biotechnology, Technical University of Munich (TUM), TUM School of Natural Sciences, Lichtenbergstr. 4, 85748 Garching, Germany.

^*^Correspondence: Thomas Brück, Werner Siemens-Chair of Synthetic Biotechnology, Technical University of Munich (TUM), TUM School of Natural Sciences, Lichtenbergstr. 4, 85748 Garching, Germany; Phone: +49 89 289 13250; Email: brueck@tum.de

## Supporting Information

| **Table S1.** DoE parameters and corresponding response variables after 96 h of cultivation for model set-up | | | | | | | | |
| --- | --- | --- | --- | --- | --- | --- | --- | --- |
| Run | A: T  [°C] | B: pH  [-] | C: DO  [%] | DCW  [g/L] | Lipid titer  [g/L] | Lipid titer  (oleate)  [g/L] | Fatty acids  (saturated)  [% (w/w)] | Fatty acids  (unsaturated)  [% (w/w)] |
| 1 | 25 | 6.5 | 30 | 42.3 | 33.8 | 17.1 | 44.4 | 55.6 |
| 2 | 25 | 6.5 | 30 | 42.6 | 36.2 | 18.3 | 44.3 | 55.7 |
| 3 | 25 | 6.5 | 30 | 39.9 | 35.8 | 18.3 | 43.8 | 56.2 |
| 4 | 25 | 6.5 | 30 | 39.0 | 32.7 | 16.0 | 45.8 | 54.2 |
| 5 | 30 | 6.5 | 10 | 43.7 | 34.7 | 16.2 | 49.0 | 51.0 |
| 6 | 20 | 6.5 | 10 | 33.2 | 24.8 | 13.4 | 40.1 | 59.9 |
| 7 | 20 | 6.5 | 50 | 35.0 | 23.0 | 11.9 | 42.0 | 58.0 |
| 8 | 20 | 7.5 | 30 | 31.5 | 26.1 | 14.0 | 40.8 | 59.2 |
| 9 | 25 | 5.5 | 10 | 46.2 | 32.2 | 17.0 | 41.2 | 58.8 |
| 10 | 25 | 5.5 | 50 | 42.2 | 30.2 | 15.5 | 43.4 | 56.6 |
| 11 | 30 | 5.5 | 30 | 41.7 | 33.9 | 16.1 | 47.4 | 52.6 |
| 12 | 20 | 5.5 | 30 | 42.3 | 30.9 | 18.0 | 36.1 | 63.9 |
| 13 | 25 | 7.5 | 10 | 28.2 | 24.0 | 11.9 | 44.7 | 55.3 |
| 14 | 25 | 7.5 | 50 | 28.3 | 22.6 | 11.0 | 45.3 | 54.7 |
| 15 | 20 | 6.5 | 50 | 32.7 | 21.5 | 11.5 | 40.8 | 56.5 |
| 16 | 20 | 6.5 | 10 | 31.6 | 22.5 | 12.2 | 39.4 | 60.6 |
| 17 | 20 | 5.5 | 30 | 33.9 | 23.3 | 13.4 | 36.6 | 63.4 |
| 18 | 30 | 5.5 | 30 | 45.9 | 33.0 | 16.0 | 46.8 | 53.2 |
| 19 | 30 | 6.5 | 10 | 45.0 | 32.0 | 15.5 | 47.5 | 52.5 |
| 20 | 30 | 6.5 | 50 | 40.5 | 31.9 | 14.7 | 49.4 | 50.6 |
| 21 | 30 | 7.5 | 30 | 35.0 | 24.0 | 10.8 | 50.4 | 49.6 |
| 22 | 25 | 6.5 | 30 | 38.5 | 29.6 | 14.9 | 44.7 | 55.3 |
| 23 | 25 | 6.5 | 30 | 34.8 | 25.3 | 12.4 | 45.8 | 54.2 |
| 24 | 25 | 6.5 | 30 | 46.0 | 32.1 | 16.2 | 44.6 | 55.4 |
| 25 | 25 | 5.5 | 10 | 42.1 | 27.9 | 14.5 | 42.1 | 57.9 |
| 26 | 25 | 5.5 | 50 | 44.2 | 30.6 | 15.7 | 43.3 | 56.7 |
| 27 | 25 | 6.5 | 30 | 45.1 | 32.0 | 15.9 | 45.0 | 55.0 |
| 28 | 30 | 7.5 | 30 | 36.8 | 20.4 | 9.4 | 49.5 | 50.5 |
| 29 | 25 | 6.5 | 30 | 39.4 | 30.1 | 15.0 | 45.0 | 55.0 |
| 30 | 25 | 7.5 | 50 | 32.5 | 22.4 | 10.8 | 46.3 | 53.7 |
| 31 | 30 | 6.5 | 50 | 48.7 | 36.2 | 17.4 | 48.0 | 52.0 |
| 32 | 25 | 6.5 | 30 | 38.9 | 29.4 | 14.7 | 44.7 | 55.3 |
| 33 | 25 | 7.5 | 10 | 40.0 | 28.3 | 14.7 | 43.4 | 56.6 |
| 34 | 20 | 7.5 | 30 | 26.5 | 18.7 | 9.6 | 42.2 | 57.8 |


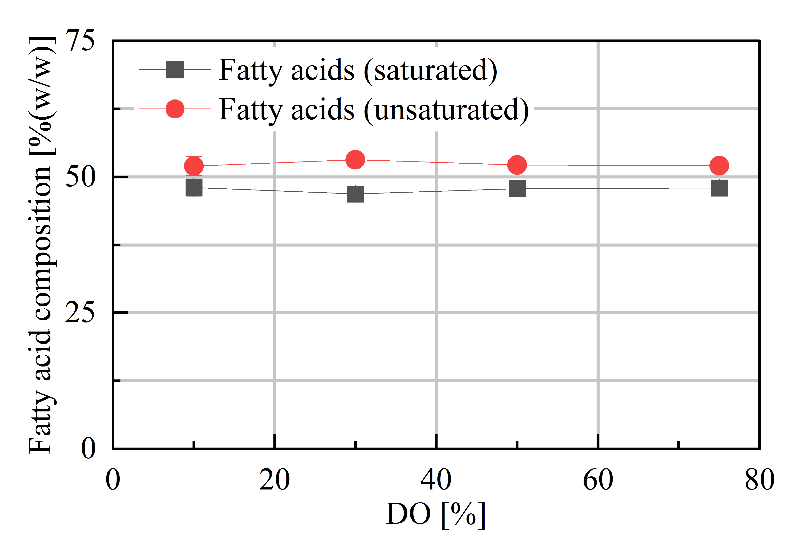


**Figure S2.** Influence of dissolved oxygen concentration on degree of fatty acid saturation. Shown are mean ± standard deviation of biological triplicates after 96 h of consumption-based acetic acid fermentation at 28°C, pH 6.5.


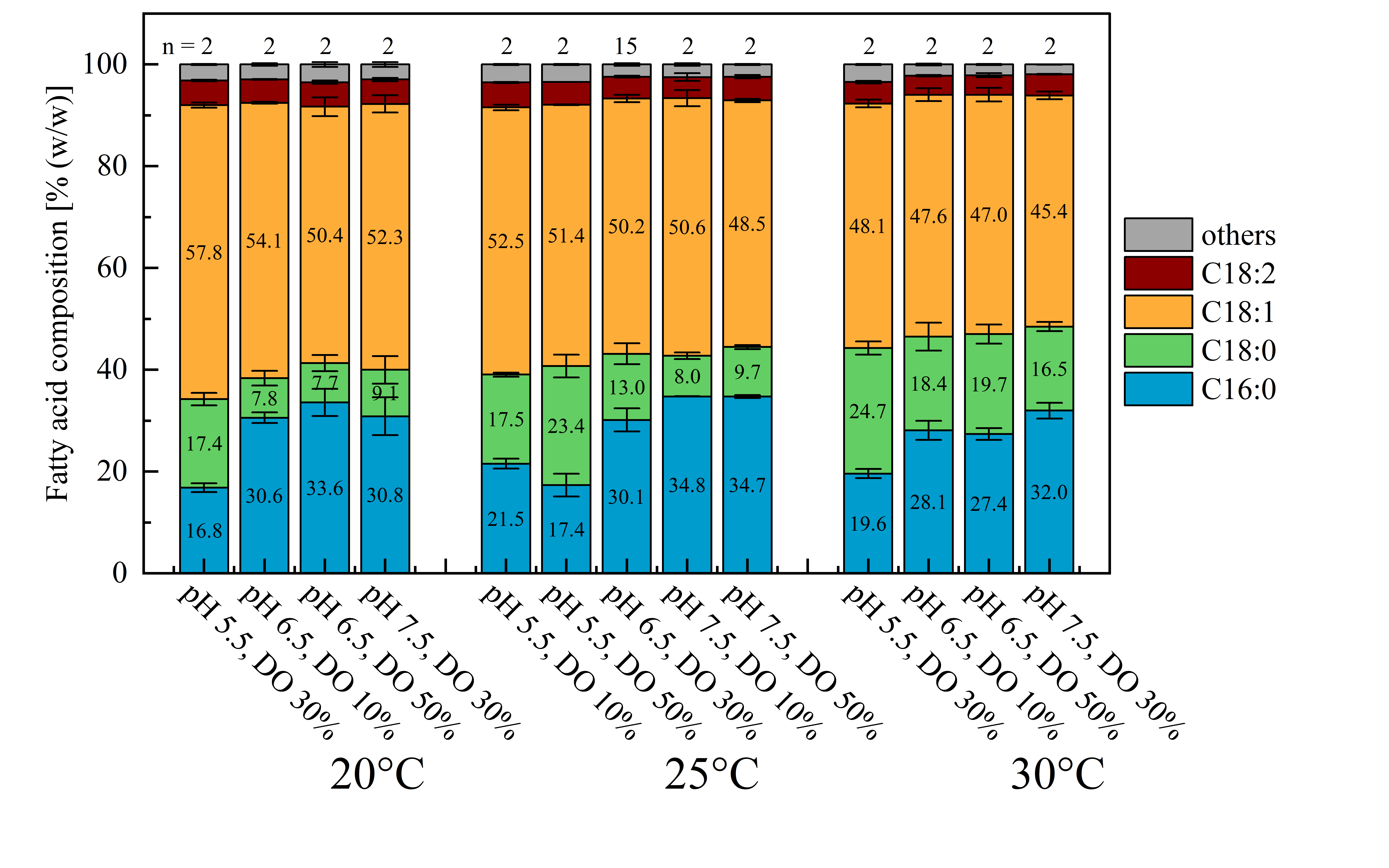


**Figure S1.** Fatty acid profiles of *C. oleaginosus* under the conditions used for BBD development. Temperature, pH, and dissolved oxygen (DO) levels varied between the experimental conditions. Shown are mean values with standard deviation of biological replicates after 96 h of consumption-based acetic acid fermentation (*n* =  2, central point *n* = 15).

| **Table S2.** Actual coefficients of quadratic response surface models | | | | | | |
| --- | --- | --- | --- | --- | --- | --- |
| *Y* | *β_0_* | *β_1_* | *β_2_* | *β_12_* | *β_11_* | *β_22_* |
| Lipid titer [g/L] | -212.03257 | 7.30816 | 47.54178 | -0.3275 | -0.089763 | -3.294080 |
| Lipid titer (oleate) [g/L] | -78.13174 | 3.14118 | 18.30395 | -0.1025 | -0.046474 | -1.374340 |
| Fatty acids (saturated) [% (w/w)] | -50.27319 | 1.91066 | 16.91678 | -0.1150 | -0.005763 | -0.956579 |
| Fatty acids (unsaturated) [% (w/w)] | 151.67204 | -1.52164 | -18.99507 | 0.1150 | -0.001342 | 1.116450 |
| Predicted responses (*Y*) are functions of temperature T [°C] and pH [-], and are described by the following quadratic regression model: $Y=\beta_{0}+\beta_{1}T+\beta_{2}pH+\beta_{12}TpH+\beta_{11}T^{2}+\beta_{22}{pH}^{2}$ | | | | | | |

**Figure S3.** Comparison of predicted and observed values for different response variables after 96 h of fermentation. Shown are linear regressions between model-predicted (abscissas) and experimentally measured values (ordinate) for (A) total lipid titer, (B) lipid titer (oleate), (C) saturated fatty acids, and (D) unsaturated fatty acids. Solid lines (—) indicate the linear fit, and corresponding regression equations and R^2^ values are provided in each subplot. In all cases, a slope of one and an intercept close to zero were obtained, which is expected in OP regressions when the predicted and observed data have similar means, as discussed in Piñeiro et al. [35].


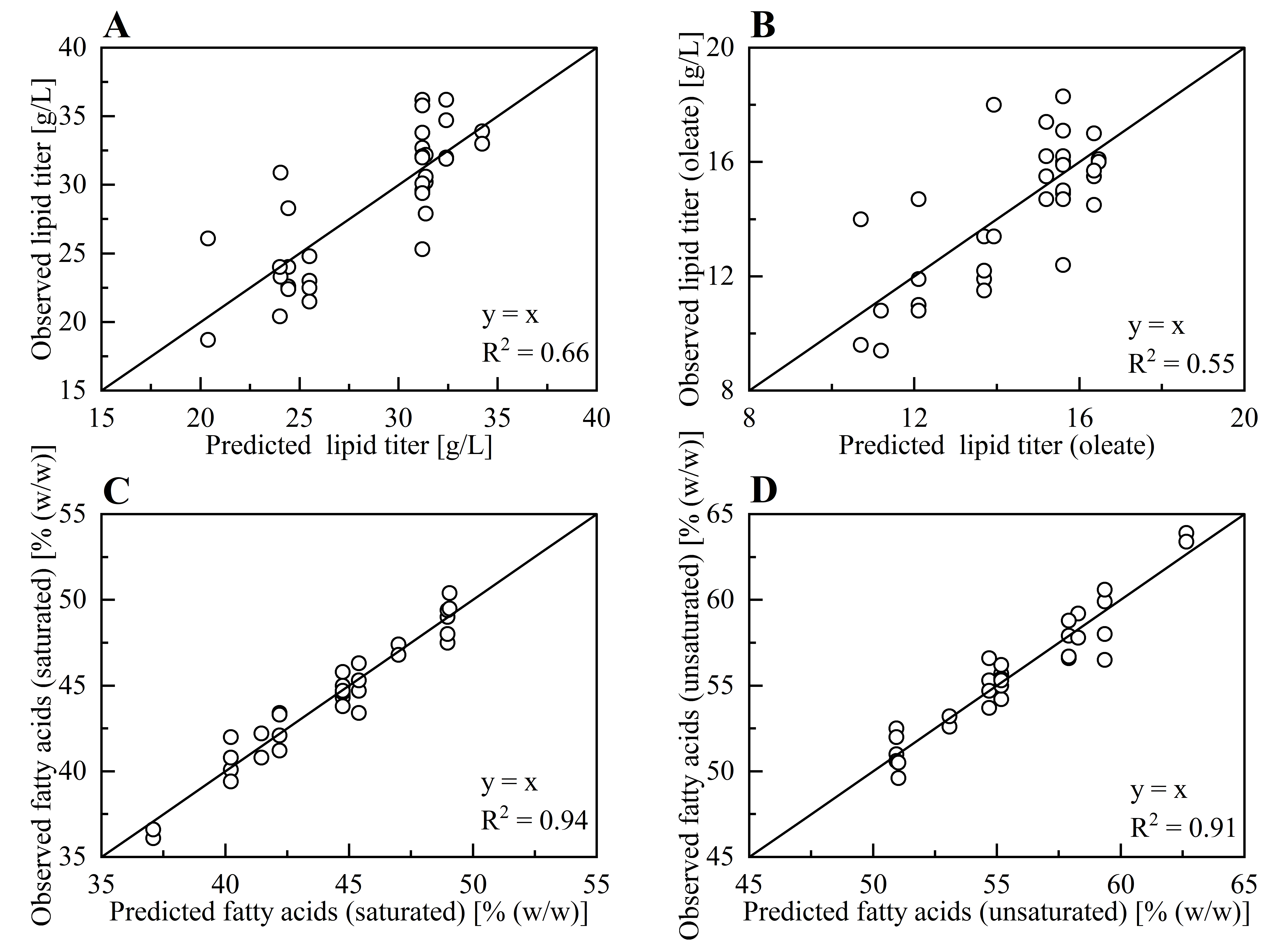


| **Table S3.** DoE parameters and corresponding response variables after 96 h cultivation for model validation | | | | | | | | |
| --- | --- | --- | --- | --- | --- | --- | --- | --- |
| Run | A: T  [°C] | B: pH  [-] | C: DO  [%] | DCW  [g/L] | Lipid titer  [g/L] | Lipid titer  (oleate)  [g/L] | Fatty acids  (saturated)  [% (w/w)] | Fatty acids  (unsaturated)  [% (w/w)] |
| 1 | 27.6 | 5.6 | 10 | 52.4 | 37.2 | 19.0 | 44.3 | 55.7 |
| 2 | 27.6 | 5.6 | 10 | 54.1 | 37.4 | 19.3 | 43.6 | 56.4 |
| 3 | 27.6 | 5.6 | 10 | 48.0 | 34.2 | 17.0 | 45.0 | 55.0 |
| 4 | 30.0 | 7.0 | 10 | 42.0 | 29.4 | 14.1 | 47.7 | 52.3 |
| 5 | 30.0 | 7.0 | 10 | 60.6 | 48.7 | 24.5 | 45.7 | 54.3 |
| 6 | 30.0 | 7.0 | 10 | 45.3 | 33.4 | 16.1 | 47.7 | 52.3 |
| 7 | 20.0 | 5.5 | 10 | 42.4 | 28.7 | 16.6 | 36.7 | 63.3 |
| 8 | 20.0 | 5.5 | 10 | 36.0 | 25.9 | 14.8 | 36.7 | 63.3 |
| 9 | 20.0 | 5.5 | 10 | 39.6 | 28.0 | 16.1 | 36.5 | 63.5 |
| 10 | 25.0 | 6.5 | 30 | 36.8 | 24.2 | 12.1 | 44.6 | 55.4 |
| 11 | 25.0 | 6.5 | 30 | 47.3 | 33.4 | 16.9 | 44.4 | 55.6 |
| 12 | 25.0 | 6.5 | 30 | 37.0 | 27.5 | 13.6 | 45.4 | 54.6 |
| 13 | 25.0 | 6.5 | 30 | 44.0 | 29.4 | 14.8 | 44.8 | 55.2 |
| 14 | 25.0 | 6.5 | 30 | 41.9 | 31.4 | 16.2 | 43.4 | 56.6 |
| 15 | 30.0 | 5.7 | 10 | 51.8 | 33.2 | 16.4 | 46.1 | 53.9 |
| 16 | 30.0 | 5.7 | 10 | 49.3 | 32.5 | 16.2 | 45.2 | 54.8 |
| 17 | 30.0 | 5.7 | 10 | 44.3 | 30.6 | 15.2 | 45.6 | 54.4 |

| **Table S4.** Validation of response surface models for predicting the responses *Y* | | | | |
| --- | --- | --- | --- | --- |
| *Y* | Within 95% PI [%] | Mean deviation [%] | Median deviation [%] | |
| Lipid titer [g/L] | 88.2 | 12.9 | 10.8 |  |
| Lipid titer (oleate) [g/L] | 94.1 | 14.0 | 8.3 |  |
| Fatty acids (saturated) [% (w/w)] | 88.2 | 2.4 | 1.7 |  |
| Fatty acids (unsaturated) [% (w/w)] | 94.1 | 2.1 | 1.4 |  |
| *n* = 17 Fermentation runs were performed for model validation. | | | |  |
